# Increased beta synchronization underlies perception-action hyperbinding in functional movement disorders

**DOI:** 10.1093/braincomms/fcae301

**Published:** 2024-10-09

**Authors:** Bernhard Pastötter, Anne Weissbach, Adam Takacs, Josephine Moyé, Julius Verrel, Fabian Chwolka, Julia Friedrich, Theresa Paulus, Simone Zittel, Tobias Bäumer, Christian Frings, Christian Beste, Alexander Münchau

**Affiliations:** Department of Cognitive Psychology, University of Trier, 54296 Trier, Germany; Institute for Cognitive and Affective Neuroscience (ICAN), University of Trier, 54296 Trier, Germany; Institute of Systems Motor Science, CBBM, University of Lübeck, 23562 Lübeck, Germany; Cognitive Neurophysiology, Department of Child and Adolescent Psychiatry, Faculty of Medicine, TU Dresden, 01307 Dresden, Germany; Institute of Systems Motor Science, CBBM, University of Lübeck, 23562 Lübeck, Germany; Institute of Systems Motor Science, CBBM, University of Lübeck, 23562 Lübeck, Germany; Institute of Systems Motor Science, CBBM, University of Lübeck, 23562 Lübeck, Germany; Institute of Systems Motor Science, CBBM, University of Lübeck, 23562 Lübeck, Germany; Institute of Systems Motor Science, CBBM, University of Lübeck, 23562 Lübeck, Germany; Department of Neurology, University of Lübeck, 23562 Lübeck, Germany; Department of Neurology, University Medical Center Hamburg-Eppendorf, 20251 Hamburg, Germany; Institute of Systems Motor Science, CBBM, University of Lübeck, 23562 Lübeck, Germany; Department of Cognitive Psychology, University of Trier, 54296 Trier, Germany; Institute for Cognitive and Affective Neuroscience (ICAN), University of Trier, 54296 Trier, Germany; Cognitive Neurophysiology, Department of Child and Adolescent Psychiatry, Faculty of Medicine, TU Dresden, 01307 Dresden, Germany; Institute of Systems Motor Science, CBBM, University of Lübeck, 23562 Lübeck, Germany

**Keywords:** functional movement disorder, post-movement beta oscillations, perception-action integration, theory of event coding, binding and retrieval in action control

## Abstract

Functional movement disorders are amongst the most common and disabling neurological conditions, placing a significant burden on the healthcare system. Despite the frequency and importance of functional movement disorders, our understanding of the underlying pathophysiology is limited, hindering the development of causal treatment options. Traditionally, functional movement disorders were considered as a psychiatric condition, associated with involuntary movements triggered by psychological stressors. Recent neurophysiological studies have unveiled cognitive alterations in affected individuals, suggesting that functional movement disorders might be better characterized by overarching neural principles governing cognitive functions. For instance, recent research has shown that the retrieval of stimulus-response bindings is altered in patients with functional movement disorders. Building upon these recent findings, our study delves into whether the initial integration of stimulus and response information is also disrupted in patients with functional movement disorders. To accomplish this, we reanalysed previously collected EEG data using refined analysis methods that provide insights into oscillatory activity and functional neuroanatomy associated with the integration of stimulus-response bindings. Our results demonstrate that post-movement beta synchronization (i) predicts behavioural stimulus-response binding and (ii) is significantly increased in patients with functional movement disorders compared to healthy controls. Utilizing beamformer analysis, we localized the difference effect to a cluster centred around the left supplementary motor area and the correlation effect to the right supplementary motor area. Extending beyond recent research that focused on the retrieval of stimulus-response bindings, our present findings reveal that the integration of stimulus and response information is already impaired in patients with functional movement disorders. These results uncover a phenomenon of hyperbinding between perception and action, which may represent a fundamental mechanism contributing to the movement impairments in patients with functional movement disorders.

## Teaser

Pastötter *et al*. reveal altered integration of stimulus and response information in patients with functional movement disorders using EEG data analysis. Compared to healthy controls they find heightened post-movement beta synchronization predicting increased stimulus-response binding, particularly in the supplementary motor area confirming altered perception-action integration in these patients.

## Graphical Abstract

**Graphical Abstract:**
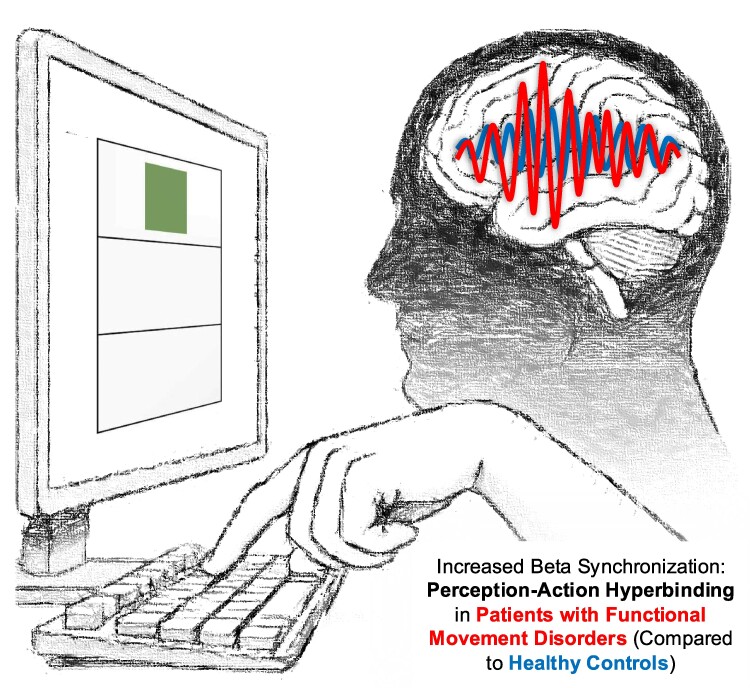

See Marthe Meppelink, M. de Jong and Beudel (https://doi.org/10.1093/braincomms/fcae339) for a scientific commentary on this article.

## Introduction

Functional movement disorders (FMD) are one of the largest groups of functional neurological disorders and among the most prevalent neurological conditions and can be highly debilitating, with more than one-third of affected patients being unable to work and requiring disability pension. One reason for this devastating scenario is a lack of knowledge on pathophysiological backgrounds hindering the development of causal treatment options. Traditionally, FMD has been classified as a dissociative disorder where psychological stressors cause involuntary movements.^1,2^ However, recent scientific research has challenged this concept,^3-5^ revealing alterations in sensory evidence accumulation processes and the integration of sensory information with motor processes. Until recently, such findings have primarily been interpreted along a predictive coding framework of FMD,^6^ which proposes a down-weighting of ‘bottom-up’ sensory information in favour of ‘top-down’ predictions (prior beliefs, feedforward information). This reduces the quality and relevance of sensory information (feedback information) available to FMD patients.

Nevertheless, it remains unclear whether these altered sensory processing mechanisms directly affect higher-level cognitive functions involved in the control of goal-directed behaviour in FMD patients. This aspect can be explored within the framework of the theory of event coding,^7,8^ which suggests that sensory (perceptual) and motor processes are represented in common codes (referred to as event files) and that such common coding reflects a means how perception and action become integrated for goal-directed actions. Utilizing the theory of event coding framework, a recent study by Weissbach *et al*.^9^ employed the EEG and event-related potential analysis to demonstrate significant deviations in processes related to the retrieval (or reactivation) of stimulus-response bindings in FMD patients compared to healthy controls (see also related event-related potential studies examining the retrieval of stimulus-response bindings in healthy controls).^10-13^ Indeed, there is broad agreement that it is important to distinguish between mechanisms governing the initial integration of perception and action and those influencing the retrieval of previously integrated perception-action codes, as suggested by the Binding and Retrieval of Action Control framework.^14^ While the study by Weissbach *et al*.^9^ suggests that the retrieval of stimulus-response bindings is altered in FMD patients, it is currently unclear whether the initial integration of perceptual and motor codes is also affected. Additionally, the underlying neurophysiological processes and functional neuroanatomy remain uncertain.

These central questions are the focal points of our current study, which involves the reanalysis of the pre-processed EEG dataset from Weissbach *et al*.^9^ with a specific focus on oscillatory activities potentially related to action control, as recently illuminated by Beste *et al*.^15^ In fact, distinct oscillatory activity profiles in theta, beta and alpha band activities may be useful to disentangle the relative contributions of different processes involved in the dynamic management of perception and action.^15^ In particular, recent research has offered compelling evidence that post-movement beta synchronization is linked to the (dis)integration of perception-action bindings (i.e. event files): Pastötter *et al*.^16^ demonstrated that post-movement beta synchronization following the response to a first-to-be integrated stimulus can predict subsequent behavioural binding effects when responding to a probe display that follows shortly thereafter. Against this background, we postulate that if event file integration is indeed stronger in FMD patients, this should be reflected as a relative increase in post-movement beta synchronization following prime responses. Furthermore, this effect might correlate with subsequent behavioural stimulus-response binding effects during probe responses. The present study is dedicated to investigating these hypotheses and elucidating the neural substrates underpinning these effects. To this end, we have also employed beamformer analysis as a tool to pinpoint the specific brain regions where group differences in post-movement beta synchronization are localized.

## Materials and methods

### Participants

Twenty-one patients diagnosed with FMD (*N* = 14 females, mean age: 39 years, age range: 16–63 years) were age and sex pairwise-matched (±6 years) to 21 healthy controls (*N* = 14 females, mean age: 40 years, age range: 16–59 years). Patients received clinical diagnosis following established diagnostic criteria.^17^ Using a standardized video-instructed and video-recorded protocol based on the Simplified Functional Movement Disorders Rating Scale (S-FMDRS),^18^ we identified 15 patients with functional gait disorders, 8 patients with functional tremor (which all had additional gait disorders), 5 patients with functional tics and 1 patient with functional jerks. The mean disorder duration was 4.43 ± 3.94 years (range: 1–12 years) and the mean S-FMDRS score was 9.24 ± 6.05 (range: 0–26). For detailed clinical data, please refer to the supplementary material of Weissbach *et al*.^9^ Written informed consent was obtained from all participants according to the Declaration of Helsinki. The study was approved by the local ethics committee of the University of Lübeck, Germany (20-136).

### Experimental task

**Figure 1:**
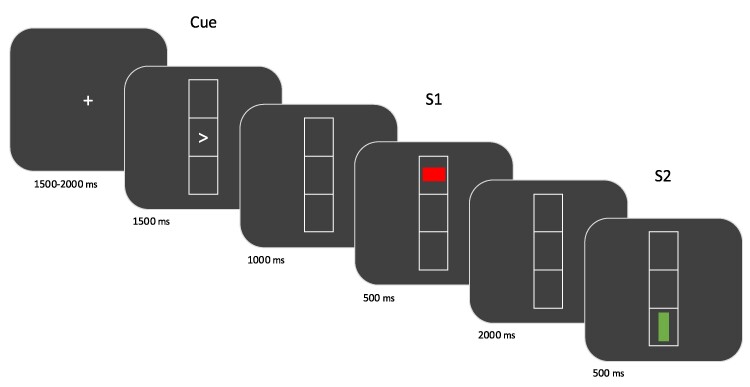
**S1R1-S2R2 paradigm.** Schematic illustration of the trial structure and the timing of single displays. Participants were instructed to provide their first response (R1) after the appearance of the first stimulus (S1; e.g. a red bar with horizontal orientation in the upper square) and their second response (R2) after the onset of the second stimulus (S2; e.g. a green bar with vertical orientation in the lower square). R1 corresponded to arrowhead’s direction in the cue display, while R2 was determined by the orientation of the bar in S2. The EEG analysis focused on the spatial analysis of beta power in the interval between R1 and S2.

The experiment utilized a modified stimulus-response paradigm.^19^ A schematic illustration of the paradigm and the exact timing of the single displays is shown in Fig. 1. At the start of each trial, a rectangle comprising three vertically aligned squares was displayed on the screen. Each trial involved two responses, R1 and R2, both requiring either a left-hand or a right-hand button press. R1 was triggered by a cue (left or right arrowhead in the middle square requiring a left-hand or a right-hand response, respectively) that preceded the first stimulus (S1). Participants were instructed not to respond immediately to the cue but to carry out R1 as soon as S1 occurred. In contrast to R1, R2 had to be carried out immediately and depending on the orientation of the bar in S2. Participants were instructed to press the left button if the bar’s orientation in S2 was horizontal and to press the right button if the orientation of the bar in S2 was vertical. Speed and accuracy were stressed for both R1 and R2.

Trials varied with repeated or alternated stimuli and responses, involving full (same position, orientation and colour of S1 and S2) or zero feature overlap (different position, orientation and colour) between S1 and S2, i.e. stimulus repetition (full overlap) or alternation (zero overlap). In addition, there was either response repetition or response alternation within trials. The main experiment consisted of 192 trials, with 48 trials for each of the 4 possible combinations of the 2 levels (repetition, alternation) of the 2 factors (stimulus dimension, response dimension). Response accuracy and mean reaction times were assessed for each participant. Before the main experiment, participants underwent a short practice block (12 trials).

### EEG data recording and pre-processing

EEG data were recorded using 60 Ag/AgCl electrodes positioned equidistantly. The sampling rate was set at 500 Hz and electrode impedances were consistently below 5 kΩ. The data were down-sampled to 256 Hz, subjected to band-pass filtering (infinite impulse response filter: 0.5–40 Hz, order of 8) and re-referenced offline to the average reference. To remove artefacts arising from eye movements, blinks and pulse, independent component analysis (infomax algorithm) was applied. Segmentation of the pre-processed data was based on the onset of R1, resulting in epochs ranging from −1500 to 3000 ms around R1. Only trials with correct responses were included in this segmentation process, while epochs containing artefacts were excluded from the subsequent analysis. It is worth noting that, due to heavy artefacts, three participants in the FMD patient group and two participants in the healthy control group were excluded from further EEG data analysis. Epochs were separately created for four different conditions: stimulus repetition with response repetition (S_rep_R_rep_), stimulus alternation with response repetition (S_alt_R_rep_), stimulus repetition with response alternation (S_rep_R_alt_) and stimulus alternation with response alternation (S_alt_R_alt_). Segmented EEG data were transformed into the time-frequency domain using a complex demodulation algorithm, which is implemented in BESA Research (v7.1).^20^ Time resolution was set to 78.8 ms (full power width at half maximum) and frequency resolution was set to 1.42 Hz (full power width at half maximum). Time-frequency data were exported in time bins of 50 ms and frequency steps of 1 Hz (from 2 to 30 Hz). Event-related power changes, time-locked to R1, were determined by calculating the temporal-spectral evolution, i.e. power changes for all time-frequency points with power increases or decreases at time point *t* and frequency *f* related to mean power at frequency *f* over a preceding baseline interval.^21^ The baseline was set from −750 to −250 ms before R1. Percent power increase indicates event-related synchronization (ERS), whereas percent power decrease indicates event-related desynchronization (ERD).^22^ For each participant, ERS/ERD values were averaged across epochs separately for the four conditions.

### Statistical analyses

Following the established approach in the S–R binding literature, we analysed the behavioural data by calculating individual binding scores. These scores were determined by subtracting the individual stimulus dimension effect in response-alternation trials (S_alt_R_alt_–S_rep_R_alt_) from the individual stimulus dimension effect in response-repetition trials (S_alt_R_rep_–S_rep_R_rep_). This calculation was performed separately for response accuracy, reaction time and the balanced integration score (BIS), which assigns equal weight to both reaction time (RT) and accuracy.^23^ The analysis of behavioural binding scores was limited to participants who were also included in the EEG analysis. The data were analysed with JASP software (version 0.18.1).^24^ For more in-depth information regarding the behavioural results, please refer to Weissbach *et al*.^9^

For the EEG scalp level analysis, we adopted a two-step approach, initially conducting non-spatial analysis, and subsequently, spatial analysis, as outlined in Pastötter *et al*.^16^ In the first step, a non-spatial cluster analysis was performed by averaging ERS/ERD spectrograms across all 60 electrodes and comparing them between FMD patients and healthy controls. In the second step, spatial topographies of clustered time-frequency effects were identified. In the non-spatial analysis, unpaired *t*-tests were computed for all time-frequency data points from 0 to 2000 ms and 2 to 30 Hz. The test statistic was calculated as the sum of t-values for adjacent time-frequency points that fell below a *P*-value of 0.05 in the *t*-test. We conducted random-permutation cluster analysis^24^ with 5000 randomization runs using BESA Statistics (v2.1, BESA Software, Gräfelfing, Germany). In each randomization run, time-frequency data for each participant were randomly assigned either to the FMD patient group (*n* = 18) or the control group (*n* = 19), and *t*-tests were calculated for each time-frequency point. At the end of each run, *t*-values for adjacent time-frequency points that fell below a *P*-value of 0.05 were summed, and the cluster with the highest sum of t-values was retained. This process generates a null distribution of cluster sums from the permutation runs, and the critical *P*_rand_ value for empirically derived time-frequency clusters was determined. Empirical clusters with a *P*_rand_ value below 0.05 went into spatial analysis, in which power changes were averaged across data points of a cluster's maximum time range and maximum frequency range, separately for each electrode. Unpaired *t*-tests comparing FMD patients and healthy controls were conducted for all electrodes. Spatial topographies were identified based on neighbouring electrodes that exhibited a *P*-value of below 0.01 in the *t*-test. Spatial clusters with at least two neighbouring electrodes were considered. The critical *P*_rand_ was calculated based on 5000 randomization runs using BESA Statistics.

The Multiple Source Beamformer, which is implemented in BESA Research (v7.1), was used to localize the neural sources of scalp-ERS/ERD difference effects between groups and brain–behaviour correlation effects. The same 500-ms baseline interval as in the scalp analysis was used. The time-frequency ranges were based on the results from the scalp-ERS/ERD group-difference cluster analysis.^25^ The frequency range was set to the scalp-ERS/ERD cluster's maximum frequency range. Because the Multiple Source Beamformer needs the same duration of the baseline interval and the target interval for reliable source-ERS/ERD calculation, the time interval for beamformer analysis was set to be a multiple of 500 ms, and Multiple Source Beamformer calculations for neighbouring target intervals were averaged, respectively. Cluster analyses of source-ERS/ERD values were done with BESA Statistics (v2.1). Group-difference clusters were identified by considering adjacent voxels that fell below a *P*-value of 0.01 in the unpaired *t*-test, which compared FMD patients with healthy controls. Brain–behaviour (product-moment) correlation clusters were calculated with a critical *P*-value of 0.01 in the *t*-test, respectively. The cluster test statistic was the sum of *t*-values of all significant voxels. The critical *P*_rand_ value was calculated based on 5000 randomization runs. Anatomic labelling of neural sources was feasible using the MNI 2 Talairach Converter with Brodmann Areas (v1.4).^26^

## Results

**Figure 2:**
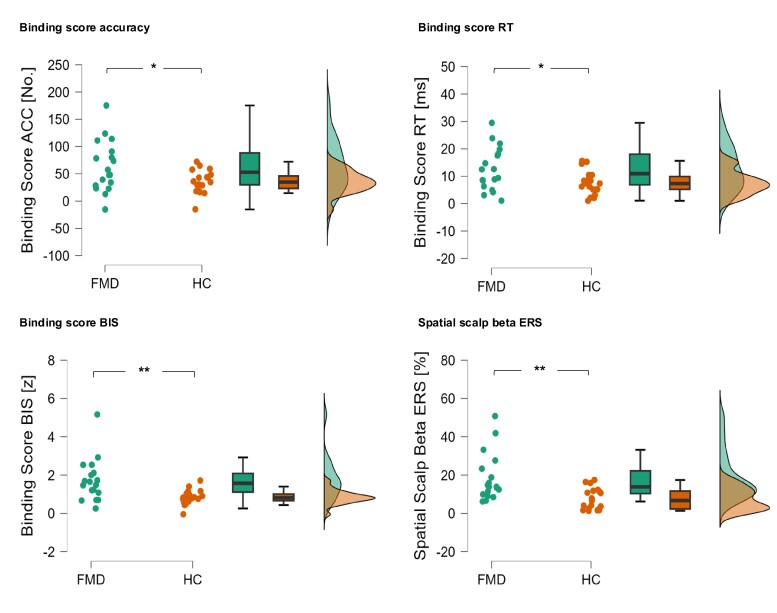
**Behavioural data and post-movement beta ERS: raincloud, boxplot and density plots.** Behavioural binding scores were computed for response accuracy (ACC), RT and the BIS, separately for patients with FMD and healthy controls (HC), as outlined in the methods section. The data for post-movement beta synchronization following R1 is plotted for the spatial scalp cluster depicted in Fig. 3. All group differences (*N* = 37) were significant: ACC, *t*(35) = 2.39, *P* = 0.022, RT, *t*(35) = 2.40, *P* = 0.022, BIS, *t*(35) = 3.28, *P* = 0.002, spatial scalp beta ERS, sum *t* value = 23.80, *P*_rand_ = 0.004 (random-permutation cluster analysis). *P < 0.05, **P < 0.01.

Patients with FMD exhibited significantly increased S–R binding across all three behavioural measures (see Fig. 2): response accuracy, *t*(35) = 2.39, *P* = 0.022, *d* = 0.79, reaction time, *t*(35) = 2.40, *P* = 0.022, *d* = 0.79, and the BIS, *t*(35) = 2.38, *P* = 0.022, *d* = 1.08. It is important to note that these findings were previously published by Weissbach *et al*.^9^ on the entire subject sample. In the present study, we focused specifically on the participants included in the EEG analysis, which confirmed hyperbinding in FMD patients in all three behavioural binding scores. Further exploratory analysis uncovered no significant correlations between the 18 patients’ S-FMDRS score and the behavioural stimulus-response binding scores: response accuracy: *r* = 0.114, *P* = 0.651, reaction time: *r* = 0.290, *P* = 0.242 and BIS: *r* = 0.336, *P* = 0.173. Moreover, the correlations between the 37 participants’ age and the behavioural stimulus-response binding scores were not significant: response accuracy: *r* = 0.168, *P* = 0.319, reaction time: *r* = 0.126, *P* = 0.458 and BIS: *r* = 0.120, *P* = 0.481.

**Figure 3:**
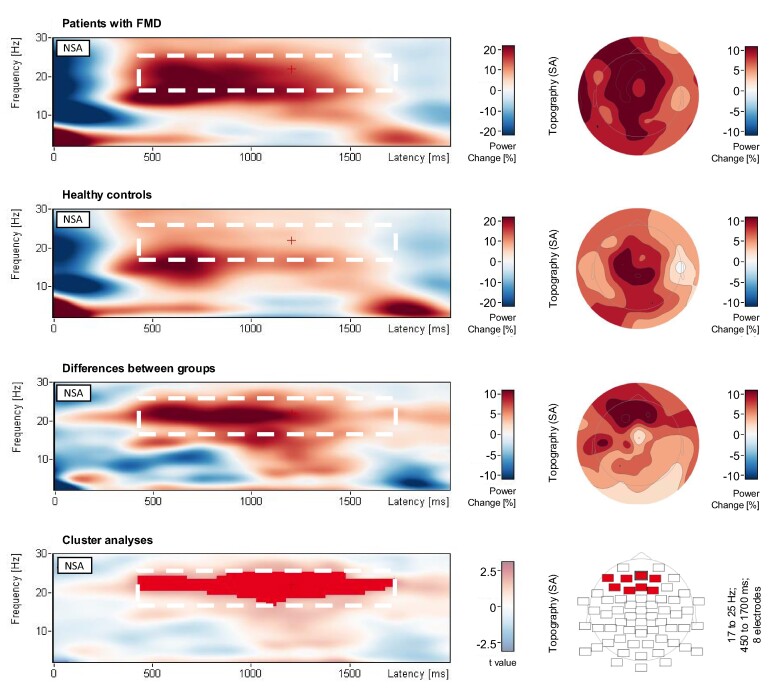
**Scalp EEG analyses.** Time-frequency spectrograms from non-spatial analysis (NSA) and topographies from spatial analysis (SA). Topographies are based on the averaged power change values from the maximum time and frequency ranges of the significant post-movement beta power cluster following R1, sum *t* value = 376.83, *P*_rand_ = 0.019 (random-permutation cluster analysis; *N* = 37). R1 onset, which is set to time point 0 on the latency axis. Cluster analysis localized the difference in post-movement beta power between groups to frontal electrode sites, sum *t* value = 23.80, *P*_rand_ = 0.004 (random-permutation cluster analysis; *N* = 37).

Regarding our primary hypothesis, which posits that event file integration is enhanced in patients with FMD, our non-spatial scalp cluster analysis unveiled a significant cluster indicating increased post-movement beta synchronization (17–25 Hz) in FMD patients when compared to healthy controls. This cluster extended from ∼450 to 1700 ms after R1, *P*_rand_ = 0.019 (Fig. 3). Spatial cluster analysis showed that this group-difference effect was significant over frontal electrode sites, *P*_rand_ = 0.004 (Fig. 3; see also Fig. 2). Remarkably, while the cluster analyses in this study encompassed the frequency range from 2 to 30 Hz, no other significant clusters were observed, neither in the theta nor in the alpha frequency range.

**Figure 4:**
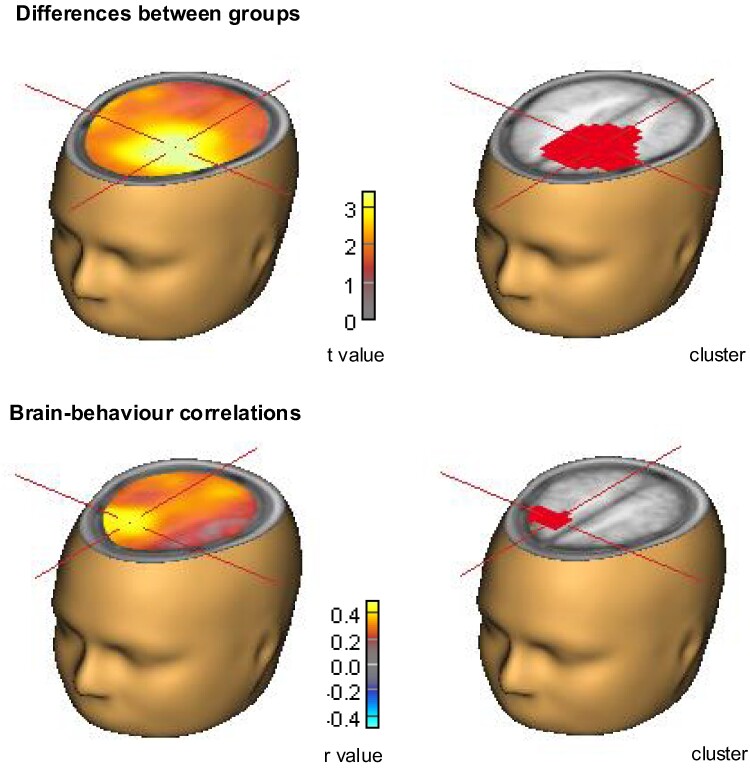
**Beamformer analyses.** Group-difference cluster analysis localized the difference in post-movement beta power between groups to a cluster centred around the left SMA, sum *t* value = 1948.20, *P*_rand_ = 0.011 (random-permutation cluster analysis; *N* = 37). Correlational analysis revealed a significant cluster in the right SMA, demonstrating a positive correlation between individual beta synchronization following R1 and the individual binding score in RT, sum *t* value = 261.44, *P*_rand_ = 0.049 (random-permutation cluster analysis; *N* = 37).

Group-difference beamformer analysis centred the group-difference effect in beta synchronization (17–25 Hz, 500–1500 ms; please note that the time interval for beamformer analysis was set to be a multiple of 500 ms; see methods section) around the left supplementary motor area (SMA), *P*_rand_ = 0.011 (see Fig. 4). Correlational beamformer analysis identified a significant cluster in the right SMA concerning RT, with larger individual RT binding scores accompanied by larger individual beta synchronization following prime responses, *P*_rand_ = 0.049 (see Fig. 4). No significant correlation clusters were observed between beta synchronization and behavioural binding scores in response accuracy, the BIS, or the S-FMDRS score. Additional exploratory analysis revealed two significant clusters showing a positive correlation between beta synchronization and participants’ age, one around the right inferior temporal gyrus, *P*_rand_ = 0.005, and one around the left SMA, *P*_rand_ = 0.024.

## Discussion

The goal of the current study was to elucidate a novel concept to frame FMD, according to which the integration of perception and action is altered in FMD patients. This concept is based on a cognitive science theorizing (i.e. theory of event coding), which is transferred to the field of neurology. While overarching principles of information processing have longer been considered in the field of FMD (e.g. predictive coding), the strength of the theory of event coding concept^8^ and its recent derivate, the Binding and Retrieval of Action Control framework^14^ is that it can capture how perception and action are dynamically managed and which neural processes are involved in this management.^15^ Especially the latter is of considerable relevance as it provides insights into the pathophysiology of FMD and hence mechanisms that are important to design treatments of these disorders.

The results of this study considerably extend our previous findings in FMD patients^9^ demonstrating that the integration of perception and action is altered in these individuals. In particular, the results show that FMD patients exhibited increased post-movement beta synchronization compared to healthy controls. Spatial scalp analysis indicated that this differential effect was significant over a frontal cluster of electrodes, for which post-movement beta synchronization additionally positively correlated with behavioural stimulus-response binding. The observed brain–behaviour correlation aligns with the findings of Pastötter *et al*.,^16^ who also reported a positive correlation between beta synchronization after R1 and the behavioural binding effect observed for the probe response (R2). It is important to highlight that while Pastötter *et al*.^16^ (using the distractor-response binding paradigm, in which all stimulus-response binding effects are due to the binding of responses to irrelevant distractor information) found this correlation for irrelevant distractor information over parietal electrode sites, in the present study (using the modified stimulus-response paradigm) the stimulus information consisted of both task-relevant (stimulus orientation) and task-irrelevant feature dimensions (colour and position) and the correlation was found over frontal sites.

Further, utilizing beamformer analysis, we localized this increased beta synchronization in FMD patients specifically to a cluster centred around the left SMA. In addition, correlational beamformer analysis revealed a significant cluster centred around the right SMA for which individual binding scores in RT were positively related to individual beta synchronization following prime responses. These localizations correspond with prior research associating the SMA with action control in healthy individuals^27^ support the idea that the present beta synchronization effects relate to the representations of stimulus-response bindings. In fact, motor-related post-movement beta power increase is typically observed less frontally in the motor cortex, namely in the precentral gyrus.^28^ Moreover, the results underscore the relevance of the SMA in the context of FMD. In fact, a previous functional MRI study using an action selection task revealed aberrant SMA activity in FMD patients compared to healthy controls, suggesting that the SMA may be a key area of motor impairment in FMD patients with excessive motor activity.^29^ Additional exploratory analysis revealed positive correlations between individual post-response beta synchronization and participant’s age, most prominent around the right inferior temporal gyrus and the left SMA. However, and more critically, no significant correlations between the behavioural stimulus-response binding scores and participant’s age were observed, indicating that stimulus-response binding effects are equally present in younger and older adults.^30^

The observed hyperbinding of perception and action in FMD patients holds substantial implications for comprehending the underlying mechanisms of these disorders. Prior research^9^ already revealed that patients with FMD show alteration when required to retrieve previously built associations between perceptual and motor codes. While these previous findings indicated that perception-action binding is a potentially new aspect of FMD, the current findings go beyond this, demonstrating that already the initial integration of stimulus and response information is changed in patients FMD. Thus, early on in the processing cascade of perceptual and motor processes, patients with FMD show alterations. This indicates that ‘hyperbinding’ of perception and action may reflect a key mechanism for a better understanding of FMD. Notably, we did not find a significant correlation between individual post-response beta synchronization and patients’ S-FMDRS scores. We posit that a larger sample size would be necessary to detect small to medium correlations, and thus, we refrain from concluding, based on the present correlation results from 18 patients, that beta synchronization lacks diagnostic value for FMD on an individual level.

It is worth noting that alterations in the processing of stimulus-response bindings are also relevant for a better understanding of other clinical conditions characterized by abnormal involuntary movements including Gilles de la Tourette (GTS).^31-33^ However, despite similar behavioural findings (increased stimulus-response binding effects regarding probe responses) in FMD and GTS, underlying neural signatures are clearly distinct. In GTS, analyses of decomposed event-related potential data revealed activation differences between patients and healthy controls related to the retrieval of stimulus-response bindings in the inferior parietal cortex,^33^ while similar analyses showed group differences between FMD patients and healthy controls primarily in motor processes with aberrant activation in the inferior frontal gyrus in individuals with FMD.^9^ These data suggest that different movement disorders may be characterized by specific neural signatures related to the retrieval of stimulus-response bindings. Clearly, further investigations into other movement disorders are required to corroborate this notion. Moreover, although both FMD and GTS seem to be conditions where altered processing of perception-action associations is a feature, in GTS it is still unclear whether the integration stimulus-response bindings is altered as well. Therefore, it is a high priority for future research to investigate post-movement beta synchronization in stimulus-response binding tasks in GTS. Additionally, it needs to be shown, in terms of both integration and retrieval of stimulus-response bindings, the extent to which FMD patients with functional tics or jerks (only six of whom were included in the present study) differ from GTS patients from a neurophysiological point of view.

An important question pertains to the implications of an increased tendency for perception-action integration in patients with FMD concerning their clinical presentation and the conceptual understanding of this disorder. Abnormalities of sensory processing relevant for the perception of movements as self-generated including sensory attenuation^34^ and temporal compression^3^ are well-documented in FMD and point towards problems in distinguishing between volitional and non-volitional movements in these patients.^4^ The increased propensity for building stimulus-response associations, which can encompass both relevant (e.g. dimension, as in the present study) and irrelevant stimulus features (e.g. colour and position, as in the present study), may indicate another significant facet of this disorder: difficulties in discerning between relevant and irrelevant stimuli and their associations with responses. For efficient goal-directed behaviour, it is important to shield intentions from competing goals to prevent premature goal shifts that can render behaviour disorganized.^35^

In other words, just as it is important to associate certain stimuli with certain responses in a given situation or context, it is crucial to abstain from forming any stimulus-response association to avoid cognitive overload. Exaggerated stimulus-response integration tendencies in FMD may lead to such overload disturbing goal-directed behaviour and causing confusion between automatic and volitional goal-directed behaviour, which is a feature in FMD.^34,36^ Therefore, a significant open question to be addressed is whether patients with FMD are generally inclined towards ‘hyperbinding’ or if this inclination is particularly pronounced when integrating irrelevant stimuli. Future studies, especially those focusing on the distractor-response binding task^16^ as opposed to the binding of responses to relevant stimuli, should explore this question.

The present findings suggest a promising avenue for further neuropsychological research into FMD and related neurological conditions. Additional studies are warranted to confirm and extend these findings. This may involve exploring techniques such as brain stimulation (e.g. alternating transcranial current stimulation in the beta frequency range over frontal sites) and biofeedback studies (e.g. regarding SMA beta activity), examining retest reliabilities of the present variables, exploring the potentials of EEG and beamformer analysis as a monitoring tool for therapeutic interventions in FMD patients, as well as investigating the broader applicability to other movement disorders. Future investigations should also delve into the clinical implications of these findings and their potential to inform therapeutic interventions aimed at improving the quality of life for FMD patients and individuals with related neurological conditions.

## Data Availability

Aggregated data (behavioural binding scores, time-frequency data and beamformer data) of all participants involved in the EEG analysis and BESA Statistics analysis codes can be accessed on OSF at https://osf.io/7ke6r/.
